# Addition of hyaluronic acid to the FIB‐4 liver fibrosis score improves prediction of incident cirrhosis and hepatocellular carcinoma in type 2 diabetes: The Edinburgh Type 2 Diabetes Study

**DOI:** 10.1002/osp4.484

**Published:** 2021-05-06

**Authors:** Sheila M. Grecian, Stela McLachlan, Jonathan A. Fallowfield, Peter C. Hayes, Indra Neil Guha, Joanne R. Morling, Stephen Glancy, Rachel M. Williamson, Rebecca M. Reynolds, Brian M. Frier, Nicola N. Zammitt, Jackie F. Price, Mark W. J. Strachan

**Affiliations:** ^1^ Usher Institute of Population Health Sciences and Informatics University of Edinburgh Edinburgh UK; ^2^ Centre for Inflammation Research Queen's Medical Research Institute University of Edinburgh Edinburgh UK; ^3^ NIHR Nottingham Biomedical Research Centre Nottingham University Hospitals NHS Trust and the University of Nottingham Nottingham UK; ^4^ Division of Epidemiology and Public Health University of Nottingham Nottingham UK; ^5^ Department of Radiology Western General Hospital Edinburgh UK; ^6^ Metabolic Unit Western General Hospital Edinburgh UK; ^7^ Metabolic Unit Borders General Hospital Melrose UK; ^8^ Centre for Cardiovascular Science Queen's Medical Research Institute University of Edinburgh Edinburgh UK; ^9^ Royal Infirmary of Edinburgh Edinburgh UK

**Keywords:** hyaluronic acid, NAFLD, risk prediction, type 2 diabetes

## Abstract

**Background:**

Type 2 diabetes (T2D) is associated with increased risk of progression to cirrhosis and hepatocellular carcinoma (HCC) in people with chronic liver diseases, particularly non‐alcoholic fatty liver disease (NAFLD). However, the absolute risk of progression is low. So, it is crucial to accurately identify patients who would benefit most from hepatology referral and intensified management. Current risk‐stratification tools are suboptimal and perform worse in people with diabetes.

**Aims:**

To determine whether the addition of complementary biomarker(s) to current NAFLD risk‐stratification tools in people with T2D could improve the identification of people who are at increased risk of developing incident cirrhosis or HCC.

**Methods:**

The Edinburgh Type 2 diabetes Study (ET2DS) is a cohort study of men and women with T2D (*n* = 1066, age 60–75 at baseline). Cases of cirrhosis and HCC were identified over 11 years of follow‐up. Biomarkers were measured at baseline and year 1 and association with incident disease was assessed using logistic regression.

**Results:**

Of existing risk‐stratification scores tested, the Fibrosis‐4 (FIB‐4) index and the AST:platelet ratio index (APRI) performed best in this cohort. Addition of hyaluronic acid (cut‐point ≥ 50 μ g/L) to FIB‐4 (cut‐point ≥ 1.3) maintained a false negative rate of ≤25% and reduced the number of people incorrectly identified as “high risk” for incident disease by ∼50%.

**Conclusions:**

The addition of hyaluronic acid to FIB‐4 reduced the proportion of people inappropriately identified as “high risk” for development of cirrhosis/HCC in a community population of otherwise asymptomatic people with T2D. These findings require a validation in independent cohorts.

## 1 INTRODUCTION

Non‐alcoholic fatty liver disease (NAFLD) is recognized as the liver component of the metabolic syndrome, a cluster of conditions including abdominal obesity, impaired glucose regulation or diabetes, hypertension, hypercholesterolemia, and hypertriglyceridemia, which are associated with increased cardiovascular risk.^1^ With rising population levels of obesity, prevalence of NAFLD is rising and 25% of people globally may be affected.^2^ Type 2 diabetes(T2D) is associated with a further increased prevalence of NAFLD, the prevalence of NAFLD steatosis being 40–70%.^3^, ^4^, ^5^, ^6^ Furthermore, people with T2D have a higher incidence of, and risk of progression to, cirrhosis and hepatocellular carcinoma (HCC).^7^, ^8^, ^9^, ^10^, ^11^

In T2D, identifying those at increased risk of developing cirrhosis/HCC is important to prompt intensified lifestyle interventions, enhanced monitoring of disease progression, and timely initiation of surveillance for varices and HCC. Screening for NAFLD in T2D is advocated in European guidelines (European Association for the Study of the Liver, European Association for the Study of Diabetes, European Association for the Study of Obesity [EASL‐EASD‐EASO]).^12^

Liver biopsy is the gold‐standard test for staging NAFLD, with histological fibrosis the most important factor predictive of disease progression in meta‐analyses.^13^, ^14^ However, biopsy is not suitable for population screening as it is an invasive procedure with a risk of serious complications. Consequently, interest in the identification of non‐invasive markers that predict those at risk of disease progression has increased. Many scores have been developed and validated in NAFLD, including the Fibrosis‐4 Index (FIB‐4), NAFLD Fibrosis Score (NFS), aspartate aminotransferase (AST):alanine aminotransferase (ALT) ratio, AST to Platelet Ratio Index (APRI), and Enhanced Liver Fibrosis (ELF) test.^15^, ^16^, ^17^, ^18^, ^19^ While these were initially developed to identify liver fibrosis at the time of testing, their ability to predict incident cirrhosis and HCC has also been validated.^11^, ^20^, ^21^, ^22^, ^23^

The performance of these scores varies between research cohorts.^20^, ^21^, ^22^, ^23^, ^24^ Typically, study populations have consisted of patients attending hepatology secondary care services and there is much less evidence to support their utility in community populations. Furthermore, these scores perform less well in people with T2D, with one study reporting that, over 4‐year follow‐up, 15% of people with diabetes with a “low‐risk” FIB‐4 score developed decompensated cirrhosis, and 17% developed HCC; by contrast, in individuals without diabetes, no participant with a “low‐risk” score developed decompensated cirrhosis or HCC.^25^ This group has reported that the use of current risk‐stratification tools would have resulted in large numbers of people who did not develop cirrhosis/HCC over 11 year follow‐up being classified as “high risk” (41% with FIB‐4), while a significant proportion (18% with FIB‐4) who did develop cirrhosis/HCC were classified as “low risk” at baseline.^26^

This study hypothesized that the addition of a complementary biomarker(s) could improve the performance of current risk‐stratification tools for the accurate identification of people with T2D who are at increased risk of developing cirrhosis or HCC.

## 2 MATERIALS AND METHODS

### 2.1 The Edinburgh Type 2 Diabetes Study

The Edinburgh Type 2 Diabetes Study (ET2DS) is a community‐based prospective cohort study of older people with T2D. Full methods have been described previously.^27^ Briefly, in 2006/2007, participants aged 60–74 with T2D were randomly selected (in age and sex bands) from the Lothian Diabetes Register (a database of almost all people with T2D living in Lothian, Scotland), and were subsequently found to be largely representative of this sampling population.^28^

Invitations to participate were sent to 5454 people, of whom 1066 (20%) attended baseline clinic. All 1066 were invited to re‐attend a clinical and liver assessment after one and four years, 939 attended the year‐1 clinic (deceased *n* = 15, unable to contact *n* = 19, unable to attend *n* = 93) and 831 the year‐4 clinic (deceased *n* = 88, unsuitable for clinical reasons *n* = 26, uncontactable *n* = 23, unable *n* = 98). All 1066 participants were followed up for outcomes until death (320 participants) or end of follow‐up in 2018.

### 2.2 Data collection‐baseline biomarker assessment

Assessments were undertaken at dedicated research clinics at the Wellcome Trust Clinical Research Facility, Western General Hospital, Edinburgh, United Kingdom, by specially trained research staff using standard operating procedures.^27^ Fasting venous blood samples were collected at baseline. Glycated hemoglobin (HbA1c), ALT, AST, alkaline phosphatase (ALP), gamma‐glutamyltransferase (γ GT), albumin, bilirubin, and platelets were analyzed using a Vitros Fusion chemistry system (Ortho Clinical Diagnostics) at the Western General Hospital. C‐reactive protein (CRP) was measured using an immunonephelometric assay; interleukin‐6 (IL‐6) and tumor necrosis factor‐alpha (TNF α) were measured using ELISA (R&D Systems), Glasgow Royal Infirmary. Hyaluronic acid was measured using a radiometric assay (Pharmacia). The Enhanced Liver Fibrosis (ELF) test was measured on fasting venous blood samples from the year‐1 clinic and analyzed using the ADVIA Centaur immunoassay system (Siemens Healthcare Diagnostics Inc.) at the iQur Laboratory.

Participants attending the year‐1 research clinics underwent a full diagnostic liver screen if serum liver enzymes or abdominal ultrasound was abnormal (including Hepatitis B and C serology, liver autoantibody titers, alpha‐fetoprotein, ferritin), and all completed standard questions on alcohol consumption (AUDIT‐C questionnaire), medication use, and past medical history. Any participant with routine liver enzyme tests above the laboratory upper limit of normal (ALT > 50 U/L, AST > 45 U/L, γ GT > 55 U/L, alkaline phosphatase (ALP) > 125 U/L), AST: ALT ratio > 1, hyaluronic acid > 100 μ g/L (in the absence of known joint disease), positive liver autoantibodies, ferritin > 1000 ng/ml, alpha‐feto protein >6 ng/ml, positive hepatitis B or C serology, spleen diameter > 13 cm, platelets < 150 × 10^9^/L (in the absence of known hematological cause), or suspected cirrhosis on ultrasound was referred for specialist hepatology review.

Fibrosis scores were calculated and cut‐point levels were used as per published work.

‐ AST to Platelet Ratio Index (APRI) was calculated as [(AST (U/L)/Upper limit normal)/platelets (×10^9^/L)] × 100.^15^
‐ AST:ALT ratio was calculated as AST (U/L)/ALT (U/L).^19^
‐ Fibrosis‐4 index (FIB‐4) was calculated as {[age (years) × AST (U/L)]/[plt (×10^9^/L) × sqrt ALT (U/L)]}.^16^, ^23^, ^29^, ^30^
‐ NAFLD Fibrosis Score (NFS) was calculated as 1.675 + (0.037 × age [years]) + (0.094 × BMI [kg/m^2^]) + (1.13 × IFG/diabetes [yes = 1, no = 0]) + (0.99× [AST (U/L]/ALT [U/L]) − (0.013 × platelet count [×10^9^/L]) − (0.66 × albumin (g/dl).^17^

### 2.3 Data collection—identification of liver disease

Possible prevalent liver disease was identified by a self‐completion questionnaire at baseline with subsequent confirmation if a clinician diagnosis was recorded in primary or secondary care medical records. Incident cirrhosis/HCC was identified and confirmed using multiple data sources, including review of all participants' hospital medical notes (TrakCare, InterSystems Corp) at 11‐year follow‐up; responses recorded in patient and GP questionnaires sent at year‐4 and year‐10 follow‐up; hospital discharge data (diagnosis and death codes) collated by ISD (Information Services Division, NHS Scotland) and collected at year‐8 follow‐up. All confirmed cases required clinician diagnosis in secondary care medical notes. Participants were identified as having “screen‐detected” cirrhosis/HCC if they were referred to hepatology following year‐1 or 4 research clinic investigation and remained under hepatology follow‐up until definitive diagnosis was made. People with prevalent cirrhosis or HCC at the baseline were excluded from analysis on incident disease.

### 2.4 Data analysis

Data were analyzed with R (R Foundation for Statistical Computing: https://www.R‐project.org/) using a complete‐case analysis. More than 5% of data were missing for all variables with the exception of ELF (*n* = 681) and ultrasound (*n* = 933). Logistic regression was used to identify the strength of association between baseline scores and biomarkers, and incident cirrhosis/HCC in this cohort. Best performing existing risk scores were chosen as the base models; assessed on performance using C‐statistic (to assess discrimination), the Hosmer–Lemeshow test for Logistic Regression (to assess calibration, >0.05 accepted) and Aikaike Information Criterion (AIC) (a measure of overall model performance). Correlation between FIB‐4 and APRI risk scores was assessed using Pearson correlation coefficient.

The strength of association of additional baseline variables that have been previously reported as potentially associated with pathogenesis or progression of liver disease was assessed. These were demographics (sex, deprivation index (SIMD), smoking status and alcohol intake), duration of T2D and HbA1c, metabolic variables (BMI, waist–hip ratio, cholesterol), markers of liver function and injury (ALP, γ GT, bilirubin, albumin and hyaluronic acid) and markers of inflammation (IL‐6, CRP and TNF ∝). Hyaluronic acid, TNF α, and γ GT were log‐transformed (natural log) to ensure linearity of response to the logit. Biomarkers that remained significantly associated with outcome after correction for markers in the base models, age and sex, were assessed individually and in combination when added to the base models using C‐statistic, AIC and Hosmer–Lemeshow test. Because of the number of cases of cirrhosis/HCC in this cohort (*n* = 43), a maximum of three additional biomarkers were added.

Due to this cohort's mixed population of screen‐detected and clinician‐diagnosed outcomes, possibly skewing time‐to‐event data as those who were screen‐detected were often diagnosed at a pre‐symptomatic stage, the primary analysis (logistic regression) did not include a time component. A sensitivity analysis was undertaken using competing risks regression to assess whether there was a significant impact of the competing risk of non‐liver death on final model performance. The Bayesian Information Criterion (BIC) was used to assess the model performance for the competing risks regression. A second sensitivity analysis was undertaken excluding any participant with definite non‐NAFLD disease.

The impact of adding the biomarkers that best improved the performance of models by AIC was assessed through calculation of sensitivity, specificity, positive predictive value (PPV), negative predictive value (NPV), false positive, and false negative rate. To undertake this, dichotomous cut‐points needed to be allocated for values of the base model and for biomarkers used. A complete‐case analysis was undertaken for model development, with only those participants with all biomarker information available included (*n* = 999, of whom 39 developed cirrhosis/HCC).

### 2.5 Ethics

Ethical permission for the study was granted by Lothian Medical Research Ethics Committee (REC reference: 16/SS/0098). All participants gave written informed consent.

## 3 RESULTS

### 3.1 Participant characteristics and incident events

Baseline characteristics are detailed in Table 1. Mean age was 67.9 years and 51.3% were male. Mean duration of T2D was 8 years, HbA1c 7.4% (57 mmol/mol), and BMI 31.4 kg/m^2^. Participants were predominantly of Caucasian ethnicity (98.3%) and 7 (0.01%) had cirrhosis/HCC. During follow‐up, 43 participants were identified with incident cirrhosis/HCC. Of these, 39 developed cirrhosis, of whom 58% developed varices, ascites, or encephalopathy. There were 13 cases of HCC (9 participants developed both cirrhosis and HCC). The etiology of incident disease was NAFLD (*n* = 31), mixed NAFLD and alcohol (*n* = 6), mixed NAFLD and α‐1 antitrypsin deficiency (*n* = 1), alcohol (*n* = 2), autoimmune (*n* = 1), or no clear diagnosis (*n* = 3).

**TABLE 1:** Baseline characteristics of the study population

Baseline characteristic		ET2DS population (*n* = 1066)
Age		67.9 (4.2)
Sex (male)		547.0 (51.3)
Scottish index of multiple deprivation quintile	1 (most deprived)	12 (11.9)
	2	208 (19.5)
	3	188 (17.6)
	4	194 (18.2)
	5 (least deprived)	349 (32.7)
Duration T2DM (years)		8.1 (6.5)
HbA1c (%)		7.4 (1.1)
HbA1c (mmol/mol)		57.0 (12.0)
BMI (kg/m^2^)		31.4 (5.7)
Smoker (current)		154.0 (14.4)
Alcohol (excess)^a^		207.0 (19.9)

*Note:* Values are mean (sd) or *n* (%).

Abbreviations: BMI, body mass index; HbA1c, glycated hemoglobin; T2DM Type 2 diabetes.

^a^ Defined as females > 14 units/week, males > 21 units/week or patient disclosed history of a current or prior alcohol problem.

### 3.2 Identification of base risk‐stratification model

The performance of five pre‐selected risk scores in the ET2DS study population is shown in Table 2. The risk scores that showed best association between score and outcome (cirrhosis/HCC) by logistic regression assessment were FIB‐4 (C‐statistic 0.86, AIC 244.5) and APRI (C‐statistic 0.85, AIC 246.5), and these were chosen as base models to assess any incremental benefit of additional biomarkers. Correlation between APRI and FIB‐4 scores in the individuals with and without incident cirrhosis/HCC was high (Pearson's *r* > 0.9).

**TABLE 2:** Odds ratios for the development of cirrhosis and HCC by fibrosis score

Fibrosis marker	Range in population	OR adjusted for age and sex (95% CI)	*p*‐Value	AIC	C‐statistic	Hosmer–Lemeshow *p*‐value
ELF	6.89–17.40	3.20 (2.18–4.84)	<0.001	195.2	0.83	<0.001
APRI	0.07–1.76	3.02 (2.37–3.94)	<0.001	246.5	0.85	0.10
AST:ALT	0.33–1.67	2.03 (1.61–2.57)	<0.001	318.4	0.73	0.03
NFS	−5.91–2.98	3.11 (2.21–4.46)	<0.001	297.3	0.80	0.001
FIB‐4	0.41–7.82	3.42 (2.60–4.62)	<0.001	244.5	0.86	0.16

*Note:* OR calculated per increase of one standard deviation in marker.

Abbreviations**:** AIC, Akaike Information Criterion; ALT, alanine aminotransferase; AST, aspartate aminotransferase; APRI, AST:platelet ratio index; CI, confidence interval; ELF, enhanced liver fibrosis; FIB‐4, Fibrosis‐4 Index; NFS, NAFLD Fibrosis Score; OR, odds ratio.

### 3.3 Association of individual biomarkers with incident cirrhosis/HCC

Individual baseline biomarkers, in addition to those already in the FIB‐4 and APRI risk scores, were assessed for their association with incident cirrhosis/HCC by odds ratio (OR) (Table 3). SIMD, HbA1c, BMI, ALP, γ GT, bilirubin, hyaluronic acid, TNF ∝, IL‐6, and CRP were associated (*p* < 0.1) in univariable analysis. SIMD, BMI, HbA1c, γ GT, hyaluronic acid, IL‐6 and CRP remained associated (*p* < 0.05) after adjustment for age, sex, and individual factors already in the base models (AST and platelets in both FIB‐4 and APRI, plus ALT and age in FIB‐4) (Table 3).

**TABLE 3:** Association of additional predictive variables with cirrhosis or HCC

Variable		Total population (*n* = 1059)	Population with cirrhosis/HCC (*n* = 43)	Population without cirrhosis/HCC (*n* = 1016)	Univariable analysis		Analysis adjusted for factors in existing models, age and sex	
					OR (95% CI)^a^	*p*	APRI (*p*)	FIB‐4 (*p*)
Age		67.9 (4.2)	68.5 (4.7)	67.9 (4.2)	1.17 (0.86–1.60)	0.31	‐	‐
Sex (male)		544 (51.4)	18 (41.9)	526 (51.8)	0.67 (0.36–1.24)	0.21	‐	‐
SIMD quintile	1 (most deprived)	125 (11.8)	9 (20.9)	116 (11.4)	3.78 (1.38–10.79)	**0.01**	**0.01**	**0.01**
	2	206 (19.5)	8 (18.6)	198 (19.5)	1.97 (0.7–5.69)	0.20	**0.03**	0.06
	3	187 (17.7)	8 (18.6)	179 (17.6)	2.18 (0.77–6.3)	0.14	0.09	0.14
	4	193 (18.2)	11 (25.6)	182 (17.9)	2.94 (1.14–8.12)	**0.03**	**0.01**	**0.02**
	5 (least deprived)	348 (32.9)	7 (16.3)	341 (33.6)				
Duration T2DM (years)		8.1 (6.5)	9.1 (6.2)	8.0 (6.5)	1.17 (0.87–1.51)	0.27	‐	‐
HbA1c (%)		7.4 (1.1)	8.1 (1.5)	7.4 (1.1)	1.53 (1.21–1.90)	**<0.001**	**<0.001**	**<0.001**
HbA1c (mmol/mol)		57 (12)	65 (16.4)	57 (12)	‐	‐	‐	‐
BMI (kg/m^2^)		31.4 (5.7)	33.7 (6.2)	31.3 (5.7)	1.44 (1.09–1.87)	**0.008**	**0.02**	**0.02**
Waist‐hip ratio		0.97 (0.1)	0.98 (0.1)	0.96 (0.1)	1.20 (0.88–1.63)	0.25	‐	‐
Smoker (current)		153 (14.4)	8 (18.6)	145 (14.3)	1.37 (0.58–2.87)	0.43	‐	‐
Alcohol (excess)^b^		204 (19.8)	13 (30.2)	191 (18.8)	1.81 (0.9–3.46)	0.08	0.73	0.71
Cholesterol (mmol/L)		4.3 (0.9)	4.2 (0.8)	4.3 (0.9)	0.84 (0.59–1.16)	0.31	‐	‐
ALT (U/L)		43.2 (14.3)	53.4 (19.9)	42.8 (13.9)	1.56 (1.26–1.94)	**<0.001**	0.07	‐
AST (U/L)		31.0 (10.4)	45.9 (15.4)	30.4 (9.7)	2.20 (1.78–2.74)	**<0.001**	‐	‐
ALP (U/L)		91.7 (27.3)	106.1 (33.5)	91.1 (26.9)	1.45 (1.15–1.82)	**0.001**	0.21	0.12
γGT (U/L)^b^		29.4 (40.3)	96.7 (86.7)	26.7 (34.7)	3.55 (2.66–4.86)	**<0.001**	**<0.001**	**<0.001**
Bilirubin (μmol//L)		10.0 (4.7)	11.2 (4.1)	9.9 (4.7)	1.24 (0.94–1.56)	0.09	0.63	0.57
Albumin (g/L)		44.8 (3.3)	44.7 (3.8)	44.8 (3.3)	0.97 (0.71–1.33)	0.86	‐	‐
Platelets (10^9^/L)		258.7 (69.3)	201.5 (77.4)	261.1 (68.0)	0.33 (0.22–0.49)	**<0.001**	‐	‐
Hyaluronic acid (μg/L)^c^		56.1 (46.6)	132.2 (85.3)	52.8 (41.3)	5.29 (3.42–8.47)	**<0.001**	**<0.001**	**<0.001**
TNF‐∝ (pg/ml)^c^		1.4 (1.5)	1.6 (0.8)	1.3 (1.5)	1.63 (1.19–2.23)	**0.002**	0.05	0.08
IL‐6 (pg/ml)		3.9 (3.5)	5.7 (3.9)	3.8 (3.5)	1.38 (1.12–1.66)	**0.001**	**0.01**	**0.02**
CRP (mg/L)		3.9 (6.0)	6.0 (8.3)	3.8 (5.9)	1.26 (1.00–1.52)	**0.03**	**0.03**	**0.03**

*Note:* Values are mean(sd) or *n* (%)

Abbreviations: γ GT, gamma‐glutamyltransferase; ALP**,** alkaline phosphatase; AST, aspartate aminotransferase; ALT, alanine aminotransferase; CRP; C‐reactive protein; HbA1c, glycated hemoglobin; IL‐6 Interleukin‐6; SIMD, Scottish Index of Multiple Deprivation, T2DM Type 2 diabetes; ; TNF**‐** ∝ tumor necrosis factor‐alpha.

^a^ Defined as females > 14 units/week, males > 21 units/week or patient disclosed history of a current or prior alcohol problem.

^b^ Results for the natural log of these values.

^c^ For continuous variables, odds ratio represents change in odds for standard deviation change in variable.

### 3.4 Addition of individual biomarkers to base prediction model

Individual biomarkers were added to the base models, and the association with incident cirrhosis/HCC was assessed using logistic regression (Table 4). Those that improved FIB‐4 model performance most in terms of AIC were HbA1c (improvement in AIC of base model from 238.2 to 228.7), hyaluronic acid (209.4), and γ GT (205.5). Hyaluronic acid and γ GT addition also showed the greatest increase in C‐statistic performance (from 0.85 to 0.89 and 0.93, respectively). For APRI, improvement in AIC was also seen most clearly with HbA1c from 243.8 to 236.2, hyaluronic acid (211.2), and γ GT (219.1), though with only modest C‐statistic improvements. When hyaluronic acid alone was added to APRI, the Hosmer–Lemeshow test was significant, indicating poor calibration.

**TABLE 4:** Performance of the baseline models (FIB‐4 and APRI) with the addition of complementary biomarkers

Model	C‐statistic	Hosmer‐Lemeshow *p*‐value	AIC
Base Model
FIB‐4	0.85	0.35	238.2
Addition of one additional variable			
FIB‐4 + HbA1c	0.87	0.43	228.7
FIB‐4 + γ GT^a^	0.93	0.98	205.5
FIB‐4 + HA^a^	0.89	0.06	209.4
FIB‐4 + BMI	0.87	0.32	232.9
FIB‐4 + SIMD	0.87	0.79	239.6
FIB‐4 + IL‐6	0.87	0.16	235.0
FIB‐4 + CRP	0.88	0.76	235.9
Mixed models			
FIB‐4, Hba1c, γ GT^a^	0.93	0.86	199.2
FIB‐4, Hba1c, HA^a^	0.90	0.10	203.5
FIB‐4, γ GT^a^, HA^a^	0.93	0.23	184.5
Full model FIB‐4, HbA1c, γ GT^a^, HA^a^	0.94	0.71	181.0
Base model			
APRI	0.85	0.92	243.8
Addition of one additional variable			
APRI + HbA1c	0.86	0.93	236.2
APRI + γ GT^a^	0.91	0.84	219.1
APRI + HA^a^	0.88	<0.01	211.2
APRI + BMI	0.86	0.52	238.6
APRI + SIMD	0.87	0.18	242.5
APRI + IL‐6	0.88	0.01	239.5
APRI + CRP	0.87	0.28	241.3
Mixed models			
APRI, Hba1c, γ GT^a^	0.91	0.84	213.6
APRI, Hba1c, HA^a^	0.89	<0.01	206.3
APRI, γ GT^a^, HA^a^	0.92	0.20	192.9
Full model APRI, HbA1c, γ GT^a^, HA^a^	0.93	0.14	189.7

Abbreviations: γ GT, gamma glutamyltransferase; AIC, Akaike Information Criterion; CRP, C‐reactive protein; HA, hyaluronic acid; HbA1c, glycated hemoglobin; IL‐6, Interleukin‐6; SIMD, Scottish Index of Multiple Deprivation.

^a^ Log‐transformed γ GT/HA variable.

Hyaluronic acid, γ GT, and HbA1c were chosen to fit to mixed models (Table 4). Regardless of the base model used, the addition of both hyaluronic acid and γ GT further improved model performance, with AIC decreasing to 184.5 (FIB‐4 as base model) or 192.9 (APRI as base model). The addition of HbA1c to either hyaluronic acid, γ GT, or both did not improve AIC or C‐statistic substantially beyond the improvement gained by hyaluronic acid and γ GT alone.

A sensitivity analysis was undertaken using a compete risk regression analysis (non‐liver death as the competing risk), which supports the finding that the addition of hyaluronic acid and/or γ GT provides the best improvement in model performance (Table 5).

**TABLE 5:** Performance of the baseline models (FIB‐4 and APRI) with the addition of complementary biomarkers, re‐run using competing risk regression analysis with non‐liver death as the competing risk

Model	BIC (null = 535.75)
Base Model	
FIB‐4	467.17
Addition of one additional variable	
FIB‐4 + HbA1c	460.27
FIB‐4 + γ GT^a^	431.97
FIB‐4 + HA^a^	445.68
FIB‐4 + BMI	469.52
FIB‐4 + SIMD	470.47
FIB‐4 + IL‐6	469.94
FIB‐4 + CRP	472.90
Mixed models	
FIB‐4, HbA1c, γ GT^a^	433.04
FIB‐4, HbA1c, HA^a^	444.16
FIB‐4, γ GT^a^, HA^a^	418.36
Full model FIB‐4, HbA1c, γ GT^a^, HA^a^	420.65
Base model	
APRI	458.58
Addition of one additional variable	
APRI + HbA1c	457.17
APRI + γ GT^a^	436.10
APRI + HA^a^	439.09
APRI + BMI	459.60
APRI + SIMD	462.82
APRI + IL‐6	460.00
APRI + CRP	463.22
Mixed models	
APRI, HbA1c, γ GT^a^	439.63
APRI, HbA1c, HA^a^	439.80
APRI, γ GT^a^, HA^a^	419.49
Full model APRI, HbA1c, γ GT^a^, HA^a^	423.29

Abbreviations: γ GT, gamma glutamyltransferase; AIC, Akaike Information Criterion; CRP, C‐reactive protein; HA, hyaluronic acid; HbA1c, glycated hemoglobin; IL‐6, Interleukin‐6; SIMD, Scottish Index of Multiple Deprivation.

^a^ log‐transformed γ GT/HA variable.

### 3.5 Predictive accuracy of the base models plus additional biomarkers

The models that performed the best according to AIC and C‐statistic (base models plus hyaluronic acid, γ GT, HbA1c, or combinations) were assessed for accuracy in the prediction of incident cirrhosis/HCC using sensitivity, specificity, PPV, NPV, false positive, and negative rates. APRI plus hyaluronic acid alone was not assessed further due to poor calibration. Cut‐points used were as follows: for FIB‐4 the “high risk” of fibrosis (>2.67), “medium to high risk” of fibrosis (≥1.3), and the “medium to high risk” adjusted for age (>2) cut‐point; for APRI, the “medium to high risk” of fibrosis (>0.5) cut‐point; for hyaluronic acid ≥100 μ g/L (appropriate for identification of fibrosis) and ≥50 μ g/L; for γ GT, the laboratory cut‐point of >55 U/L, and >20 U/L; for HbA1c > 7.5. The second lower cut‐points for hyaluronic acid and γ GT were chosen arbitrarily, with the aim of attempting to reduce false negative results.

Hyaluronic acid (cut‐point > 50 μ g/L) plus FIB‐4 (≥1.3) was the only model with a false negative rate ≤25% (*n* = 10/40), thus correctly identifying the majority of those truly at high risk at baseline (Table 6). FIB‐4 plus hyaluronic acid (cut‐point ≥ 50 μ g/L) reduced the number of people assessed as ‘high‐risk’ that did not develop cirrhosis/HCC during follow‐up (i.e., false positive rate) by 46% (399–214). Results were similar using the combined fibrosis marker as a part of the EASL‐EASD‐EASO algorithm. Using APRI as a base model, false negative rates were ≥50%.

**TABLE 6:** Predictive ability of models by sensitivity, specificity, PPV, NPV, false positives and false negatives

Model	Sens (%, 95% CI)	Spec (%, 95% CI)	PPV (%, 95% CI)	NPV (%, 95% CI)	False ^+^ve *n* (%)	False ^–^ve *n* (%)
FIB‐4 > 2.67	40 (25–57)	98 (97–99)	46 (29–63)	98 (96–98)	19 (2)	24 (60)
FIB‐4 > 2.0	62 (46–77)	92 (90–93)	23 (16–33)	98 (97–99)	82 (8)	15 (37)
FIB‐4 ≥ 1.3	82 (67–93)	59 (56–62)	8 (5–11)	99 (98–100)	399 (41)	7 (18)
As addition of further variables will increase false negative values, only FIB‐4 ≥1.3 was taken forward.						
FIB‐4 ≥ 1.3, γ GT > 55	45 (29–62)	95 (94–97)	28 (18–41)	98 (97–99)	46 (5)	22 (55)
FIB‐4 ≥ 1.3, γ GT > 20	72 (56–85)	82 (79–84)	14 (10–20)	99 (98–99)	176 (18)	11 (28)
FIB‐4 ≥ 1.3, HA ≥ 100	62 (46–77)	95 (93–96)	32 (22–44)	98 (97–99)	53 (5)	15 (38)
FIB‐4 ≥ 1.3, HA ≥ 50	75 (59‐87)	78 (75–81)	12 (8–17)	99 (98–99)	214 (22)	10 (25)
FIB4 ≥ 1.3, HbA1c > 7.5	47 (32–64)	88 (85–90)	13 (8–20)	98 (96–99)	122 (12)	21 (53)
FIB‐4 ≥ 1.3, HA ≥ 50, γ GT > 20	65 (48‐79)	90 (88–92)	22 (15–30)	98 (97–99)	94 (10)	14 (35)
FIB4 ≥ 1.3, HA ≥ 50, HbA1c > 7.5	45 (29–62)	93 (91–94)	20 (12–30)	98 (96–99)	72 (7)	22 (55)
FIB4 ≥ 1.3, γ GT > 20, HbA1c > 7.5	40 (25–57)	94 (92–95)	22 (13–33)	97 (96–98)	58 (6)	24 (60)
Fib4 ≥ 1.3, HA ≥ 50, GGT > 20, HbA1c > 7.5	38 (23–54)	97 (95–98)	31 (19–46)	97 (96–98)	33 (3)	25 (63)
APRI > 0.5	53 (36–68)	94 (93–96)	27 (18–38)	98 (97–99)	57 (6)	19 (48)
APRI > 0.5, γ GT > 55	35 (21–52)	98 (97–99)	45 (27–64)	97 (96–98)	17 (2)	26 (65)
APRI > 0.5, γ GT > 20	50 (34–66)	96 (95–97)	36 (24–50)	98 (97–99)	35 (4)	20 (50)
APRI > 0.5, HbA1c > 7.5	33 (19–49)	98 (97–99)	37 (21–55)	97 (96–98)	22 (2)	27 (68)
APRI > 0.5, HA ≥ 50, γ GT > 20	50 (34–66)	98 (96–98)	45 (30–61)	98 (97–99)	24 (2)	20 (50)
APRI > 0.5, HA ≥ 50, HbA1c > 7.5	33 (19–49)	98 (97–99)	45 (26–64)	97 (96–98)	16 (2)	27 (68)
APRI > 0.5, γ GT > 20, HbA1c > 7.5	30 (17–47)	99 (98–99)	50 (29–71)	97 (96–98)	12 (1)	28 (70)
APRI > 0.5, HA ≥ 50, GGT > 20, HbA1c > 7.5	30 (17–47)	99 (98–100)	57 (34–78)	97 (96–98)	9 (1)	28 (70)
EASL guidelines‐ USS steatosis + FIB‐4 ≥ 1.3 OR ALT > 50 OR AST > 45 OR γ GT > 55	86 (71–95)	60 (57–63)	8 (6–11)	99 (98–100)	346 (40)	5 (14)
EASL (USS) + HA ≥50	81 (64–92)	81 (78–84)	15 (10–21)	99 (98–100)	163 (19)	7 (19)
EASL guidelines‐ FLI positive + FIB‐4 ≥ 1.3 OR ALT > 50 OR AST > 45 OR γ GT > 55	90 (76–97)	58 (55–61)	8 (6–11)	99 (98–100)	411 (42)	4 (10)
EASL (FLI) + HA ≥50	78 (62–89)	79 (76–81)	13 (9–18)	99 (98–99)	206 (21)	9 (23)

Abbreviations: γ GT, gamma‐glutamyltransferase; ALT, alanine aminotransferase; AST, aspartate aminotransferase; FLI, fatty liver index; HA, hyaluronic acid; HbA1c, glycated hemoglobin; NPV, negative predictive value; PPV, positive predictive value; sens, sensitivity; spec, specificity; USS, ultrasound assessed.

Two sensitivity analyses were undertaken, one excluding participants with definite non‐NAFLD disease and another excluding participants who developed HCC in a non‐cirrhotic liver. Neither analysis materially changed the results (Tables 7 and 8).

**TABLE 7:** Predictive ability of models by sensitivity, specificity, PPV, NPV, false positives and false negatives‐final models, participants with definite non‐NAFLD disease excluded (*n* = 3)

Model	Sens (%, 95% CI)	Spec (%, 95% CI)	PPV (%, 95% CI)	NPV (%, 95% CI)	False ^+^ve *n* (%)	False ^–^ve *n* (%)
FIB‐4 ≥ 1.3	84 (68–94)	59 (56–62)	7 (5–10)	99 (98–100)	399 (41)	6 (16)
FIB‐4 ≥ 1.3, HA ≥ 50	78 (62–90)	78 (75–81)	12 (8–17)	99 (98–100)	214 (22)	8 (22)
EASL guidelines‐ USS steatosis + FIB‐4 ≥ 1.3 OR ALT > 50 OR AST > 45 OR γ GT > 55	89 (75–97)	60 (57–64)	8 (6–11)	99 (98–100)	376 (44)	4 (11)
EASL (USS)+ HA ≥50	78 (62–90)	81 (79–84)	14 (10–19)	99 (98–100)	179 (21)	8 (22)
EASL guidelines‐ FLI positive + FIB‐4 ≥ 1.3 OR ALT > 50 OR AST > 45 OR γ GT > 55	89 (75–97)	58 (55–61)	7 (5–10)	99 (98–100)	413 (43)	4 (10)
EASL (FLI) + HA ≥ 50	78 (62–90)	79 (76–81)	12 (8–17)	99 (98–100)	208 (21)	8 (22)

Abbreviations: γ GT, gamma‐glutamyltransferase; ALT, alanine aminotransferase; AST, aspartate aminotransferase; FLI, fatty liver index; HA, hyaluronic acid; NPV, negative predictive value; PPV, positive predictive value; sens, sensitivity; spec, specificity; USS, ultrasound assessed.

**TABLE 8:** Predictive ability of models by sensitivity, specificity, PPV, NPV, false positives and false negatives‐final models, participants who developed HCC in a non‐cirrhotic liver excluded (*n* = 4)

Model	Sens (%, 95% CI)	Spec (%, 95% CI)	PPV (%, 95% CI)	NPV (%, 95% CI)	False ^+^ve *n* (%)	False ^–^ve *n* (%)
FIB‐4 ≥ 1.3	84 (68–94)	59 (56–62)	7 (5–10)	99 (98–100)	394 (41)	6 (16)
FIB‐4 ≥ 1.3, HA ≥ 50	76 (59–88)	78 (75–81)	12 (8–16)	99 (98–99)	211 (22)	9 (24)
EASL guidelines‐ USS steatosis + FIB‐4 ≥ 1.3 OR ALT > 50 OR AST > 45 OR γ GT > 55	92 (78–98)	60 (57–63)	8 (6–12)	99 (98–100)	371 (40)	2 (8)
EASL (USS) + HA ≥50	92 (78–98)	67 (63–70)	10 (7–13)	100 (99–100)	315 (33)	3 (8)
EASL guidelines‐ FLI positive + FIB‐4 ≥ 1.3 OR ALT > 50 OR AST > 45 OR γ GT > 55	92 (78–98)	57 (54–61)	8 (5–11)	99 (98–100)	408 (43)	3 (8)
EASL (FLI)+ HA ≥ 50	89 (75–97)	64 (61–67)	9 (6–12)	99 (98–100)	344 (36)	4 (11)

Abbreviations: γ GT, gamma‐glutamyltransferase; ALT, alanine aminotransferase; AST, aspartate aminotransferase; FLI, fatty liver index; HA, hyaluronic acid; PPV, positive predictive value; NPV negative predictive value; sens, sensitivity; spec, specificity; USS, ultrasound assessed.

## 4 DISCUSSION

Serum hyaluronic acid in conjunction with the FIB‐4 risk‐stratification score reduced the number of false positive results in this cohort, without substantially increasing the false negative results, either in isolation or within the EASL‐EASD‐EASO algorithm. To this team's knowledge, this is the first study to examine the use of hyaluronic acid for risk stratification of liver disease in a community population with T2D.

Addition of hyaluronic acid improved the association of the FIB‐4 model with incident cirrhosis/HCC. Moreover, when hyaluronic acid (cut‐point ≥ 50 μ g/L) was added to the FIB‐4 risk‐stratification tool, the number of people inappropriately classified as “high risk” was reduced by 46% (*n* = 399 to *n* = 214), while increasing those inappropriately classified as “low risk” from 18% to 25% (*n* = 7 to *n* = 10). APRI performed similarly to FIB‐4 as a base model. Both have similar component factors and the scores were highly correlated. Therefore, the additive effect of using both markers in combination was not assessed. The addition of hyaluronic acid to APRI had poor calibration and was not assessed further in isolation. A “high risk” FIB‐4 plus hyaluronic acid score was associated with a median time‐to‐diagnosis of cirrhosis/HCC for approximately 3 years, with the majority presenting within 6 years. Due to the often asymptomatic course of NAFLD, it seems likely that a significant proportion of these individuals had undiagnosed cirrhosis at the time of the baseline assessment, while the remainder had at least advanced fibrosis.

The ET2DS specifically studied liver outcomes in a community population of otherwise‐asymptomatic individuals with T2D, who did not necessarily have liver disease. Almost all other studies have identified outcomes in cohorts recruited from secondary care hepatology clinics, with established NAFLD diagnoses and likely advanced pathology. European guidelines recommend screening in populations like the one represented by the ET2DS cohort, making this a suitable testbed for assessing the impact of potential population screening strategies.^12^ The ET2DS is a moderate‐sized cohort. Participants were well‐characterized at baseline to allow accurate determination of any potential additional baseline risk factors and were followed up using multiple sources of information to accurately identify incident disease.

There are limitations to this study. The ET2DS is a single‐center study, undertaken in people aged 60–75 years, of predominantly Caucasian origin (98.3%) and care should be taken in extrapolating results to other populations. While the etiology of incident disease was almost entirely NAFLD, cirrhosis/HCC of other etiologies was included. There are known difficulties in determining the exact contributions of different etiologies (or cofactors) in cirrhosis/HCC development; thus, the investigation of all liver diseases seemed more relevant in a real‐world setting.^31^ A sensitivity analysis that excluded participants who developed definite non‐NAFLD disease did not materially affect results. Medication exposure data were not analyzed.

The incidence data may be an underestimate as it is possible that some asymptomatic participants who developed cirrhosis/HCC during follow‐up were not identified, as screening for cirrhosis/HCC at 11‐year follow‐up was not repeated. Alternatively, incidence data may overestimate the clinical burden as a substantial proportion of diagnoses were made after hepatology referral following year‐1 and year‐4 screening investigations. NAFLD cirrhosis can have a silent natural history and may not manifest clinically for many years. Thus, some people who may never have developed overt cirrhosis, or may have died before their disease became clinically apparent may have been identified. However, 58% of those identified with cirrhosis developed varices, ascites and/or encephalopathy and 23% developed HCC, so it is likely that a large majority would have presented with clinical sequelae during follow‐up.

Those who were diagnosed following screening in year 1 may have had undiagnosed cirrhosis/HCC at the baseline. However, the study considered prevalent disease to be only that which was clinically apparent at baseline because the diagnosis of cirrhosis for some referred post‐screening came many years following that referral (people were kept under active follow‐up due to high‐risk features for progression). Additionally, the time to diagnosis for those who were diagnosed following year‐1 screening and those diagnosed following routine clinical referral significantly overlapped, suggesting that stage of disease in the two groups at baseline did not differ significantly.^26^

ELF was measured at the year‐1 clinic (all other biomarkers at baseline), so this analysis used slightly different “baseline” time points. However, this group has demonstrated previously that there is no significant difference in model performance using baseline or year‐1 data; in addition, no participant was identified with incident disease prior to the year‐1 clinics.^26^ Hyaluronic acid is known to be raised in the context of joint, as well as liver disease. As accurate data on joint disease prevalence for the whole cohort at baseline were not available, individuals with joint disease were not excluded. However, as hyaluronic acid was used in conjunction with other markers of liver fibrosis, isolated elevation of hyaluronic acid due to joint disease should not have had a material impact on the models.

This group has previously described the performance of current risk‐stratification models in predicting cirrhosis and HCC in different cohorts.^26^ In addition, risk‐stratification scores perform worse in populations with diabetes than in those without.^25^ Previous cohort studies have failed to consistently identify individual non‐invasive biomarkers that are associated NAFLD progression.^13^, ^14^ This study demonstrates that using serum hyaluronic acid in conjunction with the FIB‐4 risk‐stratification score can reduce the burden of false positive results. Hyaluronic acid is a glycosaminoglycan found in connective tissue that is almost exclusively cleared by liver metabolism. Raised levels of hyaluronic acid are known to be associated with cirrhosis.^32^ However, few studies have assessed it as a prognostic marker. In combination with other biomarkers as part of the ELF risk‐stratification tool, hyaluronic acid is associated with fibrosis in NAFLD.^18^ One study found a significant association with rising hyaluronic acid and all‐cause mortality, liver mortality and liver transplant‐free survival.^33^ Thus, the present data, finding suggesting its utility in predicting those who are at “high risk” of developing incident cirrhosis/HCC, is consistent with published data.

The present findings derive from a single moderately sized cohort and need validation in other independent cohorts. A change in FIB‐4 plus hyaluronic acid over time was not examined. Moreover, there were too few individuals who developed cirrhosis/HCC to determine reliably if the median time‐to‐diagnosis was more prolonged in those with a “low‐risk” score compared to those with a “high‐risk” score. If the time‐to‐diagnosis was more prolonged in those with a “low‐risk” score, repeat assessment at intervals of several years might successfully identify additional individuals who would develop cirrhosis/HCC.

In conclusion, the prevalence of both NAFLD and T2D are rising in association with the rising population prevalence of obesity. T2D is associated with an increased risk of cirrhosis/HCC.^8^, ^9^ As a result, both European and American guidelines advocate a high index of suspicion for liver disease in T2D, with European guidelines recommending routine screening.^12^, ^34^ However, current risk‐stratification tools perform sub‐optimally, especially in diabetes.^25^, ^26^ This study shows that using a combination of FIB‐4 and hyaluronic acid for risk‐stratification can significantly reduce false positive rates without substantially increasing false negative rates. This makes this combination a possible candidate for community screening, as it would lead to identification of a substantial proportion of cases while reducing stress on health systems from false positive results. These findings are promising, but require further validation. Furthermore, the false positive rates for the FIB‐4 and hyaluronic acid combination remain high and so it is acknowledged that better biomarkers are required for the identification of people with T2D at risk of developing cirrhosis/HCC.

## AUTHOR CONTRIBUTIONS

Sheila M. Grecian wrote the manuscript. Joanne R. Morling was principal investigator of the ET2DS, designed the study, analyzed and interpreted the data. Mark W.J. Strachan was the lead investigator of the liver sub‐study of the ET2DS, designed the study, analyzed and interpreted the data. Rebecca M. Reynolds, Brian M. Frier, Peter C. Hayes, Jonathan A. Fallowfield, Rachel M. Williamson, Indra Neil Guha, and Stephen Glancy contributed to study design. Sheila M. Grecian, Stela McLachlan, Rachel M. Williamson, and Joanne R. Morling contributed to data collection, analysis and interpretation. All authors contributed to revision and final approval of the article.

## CONFLICT OF INTERESTS

Jonathan A. Fallowfield has served as a consultant or advisory board member for Novartis, Ferring Pharmaceuticals, Macrophage Pharma, Aquilla BioMedical, Galecto Biotech, Caldan Therapeutics, Cypralis, NorthSea Therapeutics, Rallybio, Tectonic, and has received research funding from Novartis and Intercept Pharmaceuticals, outside the submitted work. Peter C. Hayes has served as speaker or on advisory boards for the following AbbVie, BMS, Eisai Ltd, Falk, Ferring, Gilead, Gore, Janssen, Lundbeck, MSD, Norgine, Novartis, ONO Pharmaceuticals, Pfizer and Roche. Joanne R. Morling reports salary support from Diabetes UK for the ET2DS. Rachel M. Williamson reported salary funding by a research grant from Pfizer for 2 years. Brian M. Frier has participated in a Speakers' Bureau for: Lilly, Novo Nordisk, MSD, Sanofi, Roche, and Abbott and has served on advisory boards for Lilly, and Zucara. Mark W.J. Strachan has received consultancy fees from Servier and Novo Nordisk and speaking fees from Napp, Sanofi, Astra Zenica, Merck and Eli Lilly. No other potential conflicts of interest relevant to this article were reported.
